# A Rapid and Sensitive Europium Nanoparticle-Based Lateral Flow Immunoassay Combined with Recombinase Polymerase Amplification for Simultaneous Detection of Three Food-Borne Pathogens

**DOI:** 10.3390/ijerph18094574

**Published:** 2021-04-26

**Authors:** Kai Chen, Biao Ma, Jiali Li, Erjing Chen, Ying Xu, Xiaoping Yu, Chuanxin Sun, Mingzhou Zhang

**Affiliations:** 1 Zhejiang Provincial Key Laboratory of Biometrology and Inspection & Quarantine, China Jiliang University, Hangzhou 310018, China; s1809071005@cjlu.edu.cn (K.C.); 16a0701109@cjlu.edu.cn (B.M.); s1709071012@cjlu.edu.cn (J.L.); s1709071002@cjlu.edu.cn (E.C.); s1809071028@cjlu.edu.cn (Y.X.); yxp@cjlu.edu.cn (X.Y.); 2 Department of Plant Biology, Uppsala BioCenter, Linnean Centre for Plant Biology, EuSwedish University of Agricultural Science (SLU), P.O. Box 7080, SE-75007 Uppsala, Sweden; Chuanxin.Sun@slu.se

**Keywords:** recombinase polymerase amplification, europium nanoparticles, lateral flow immunoassay, multiplex detection, food-borne pathogens

## Abstract

Food-borne pathogens have become an important public threat to human health. There are many kinds of pathogenic bacteria in food consumed daily. A rapid and sensitive testing method for multiple food-borne pathogens is essential. Europium nanoparticles (EuNPs) are used as fluorescent probes in lateral flow immunoassays (LFIAs) to improve sensitivity. Here, recombinase polymerase amplification (RPA) combined with fluorescent LFIA was established for the simultaneous and quantitative detection of *Listeria monocytogenes*, *Vibrio parahaemolyticus*, and *Escherichia coli* *O157:H7*. In this work, the entire experimental process could be completed in 20 min at 37 °C. The limits of detection (LODs) of EuNP-based LFIA–RPA were 9.0 colony-forming units (CFU)/mL for *Listeria monocytogenes*, 7.0 CFU/mL for *Vibrio parahaemolyticus*, and 4.0 CFU/mL for *Escherichia coli* *O157:H7*. No cross-reaction could be observed in 22 bacterial strains. The fluorescent LFIA–RPA assay exhibits high sensitivity and good specificity. Moreover, the average recovery of the three food-borne pathogens spiked in food samples was 90.9–114.2%. The experiments indicate the accuracy and reliability of the multiple fluorescent test strips. Our developed EuNP-based LFIA–RPA assay is a promising analytical tool for the rapid and simultaneous detection of multiple low concentrations of food-borne pathogens.

## 1. Introduction

Food-borne pathogens represent one of the most serious threats to human health, leading to many diseases. The diseases caused by the consumption of contaminated food ingredients are called food-borne diseases [1]. This problem is considered serious all over the world. The three most prevalent food-borne pathogens are *Listeria monocytogenes*, *Vibrio parahaemolyticus*, and *Escherichia coli O157:H7* [2,3,4]. *Listeria monocytogenes* is a rod-shaped, Gram-positive food-borne bacterium with high tolerance to different growth conditions, including high concentrations of salt and low pH levels [5]. Due to its ability to survive in harsh environments, it can continue to exist in the food supply chain even under conventional sanitary conditions, which may lead to food contamination [6]. *Vibrio parahaemolyticus* is a curved rod-shaped, Gram-negative halophilic bacterium that is mainly distributed in various seafood products, such as fish, shrimp, and shellfish [7,8,9]. Contaminated seafood usually causes food-borne poisoning, accompanied by vomiting, diarrhea, gastrointestinal cramps, and other gastroenteritis symptoms [10]. *Escherichia coli O157:H7*, an important food-borne pathogen, is often associated with life-threatening diseases [11]. *Escherichia coli O157:H7* can cause hemorrhagic colitis and the more severe complication of hemolytic uremic syndrome (HUS) [12]. It is crucial to find a fast and efficient way to simultaneously detect these three pathogens in contaminated food.

Conventional culture-based assays are the gold standard for the identification of pathogenic microorganisms and provide the most accurate results together with subsequent biochemical analysis. However, they require a lengthy and laborious process of cultivation and identification. With the development of molecular biology, polymerase chain reaction (PCR) provides a high-throughput and multiplex tool in microbial diagnosis. Traditional PCR assays are time-consuming and require large and expensive thermocyclers [13]. Recently, several isothermal nucleic acid amplification techniques were established, which avoid the above-mentioned disadvantages. In addition, the possibility of visually assessing the results has increased the use of these techniques as simple and affordable alternative molecular diagnostic tools [14].

Recombinase polymerase amplification (RPA) can simply and quickly amplify nucleic acids under isothermal conditions [15,16]. Instead of using heat to denature the target DNA strand, the RPA reaction operates isothermally by using enzymes to separate the double-stranded DNA [5]. After being incubated at 37–42 °C for 20 min, the amplified products can be detected [17]. The amplification products of RPA can be visualized by agarose gel electrophoresis, real-time fluorescent probes, and lateral flow immunoassay (LFIA) [18]. LFIA analysis requires 5–10 min to complete without any special equipment. Therefore, the combination of RPA and LFIA (LFIA–RPA) is a good choice and can further improve simplicity; the total detection time is usually less than 30 min [19].

In LFIA tests, colloidal gold (CG) nanoparticles are the most extensively used labeling material. Previous studies mostly used colloidal gold technologies, but they have the disadvantage of low sensitivity. The development of fluorescent markers has attracted attention because they can enhance the detection signal and increase sensitivity; these include europium nanoparticles (EuNPs), time-resolved fluorescent nanobeads (TRFNs), and quantum dots (QDs) [20,21,22]. However, the low analytical sensitivity and relatively poor quantitative resolution when using TRFNs and QDs limit their further application in complex samples. EuNPs represent an ideal fluorescent label. They have a long fluorescence lifetime, high fluorescence, and nontoxic effect on the extracted sample. After brief background fluorescence attenuation during sample detection, the specific fluorescence of EuNPs can be analyzed, such that background fluorescence interference can be eliminated by extending the measurement time [23]. Compared with the traditional colloidal gold-based immunochromatography method, EuNP immunochromatography can improve the limit of detection (LOD) up to 10-fold [24]. Therefore, EuNPs can be used as an excellent tool to improve sensitivity.

In this study, we report a EuNP-based LFIA–RPA assay that can simultaneously detect *Listeria monocytogenes*, *Vibrio parahaemolyticus*, and *Escherichia coli O157:H7*. Moreover, we designed and optimized EuNP-based LFIA–RPA and CG–LFIA–RPA experiments, as well as examining their analytical performance. Our experiments further promote the application of EuNP-based LFIA–RPA in food safety diagnosis.

## 2. Materials and Methods

### 2.1. Bacterial Strains and DNA Template Preparation

The strains used in this study are listed in Table 1. *Listeria monocytogenes* (ATCC19111), *Vibrio parahaemolyticus* (ATCC33847), and *Escherichia coli O157:H7* (ATCC35150) were chosen as the reference strains for all inoculation experiments of multiplex EuNP-based LFIA–RPA. *Listeria monocytogenes* strains were grown on buffered *Listeria* enrichment broth (BLEB) (Thermo Fisher Scientific Inc., Waltham, MA, USA). Vibrio parahaemolyticus strains were grown on alkaline peptone water (APW, Hopebio, Qingdao, China) supplemented with 3% NaCl. *Escherichia coli O157:H7* strains were grown on lauryl tryptose (LST) broth (Thermo Fisher Scientific Inc., Waltham, MA, USA). All strains were incubated overnight at 35 °C for 20–24 h. The bacterial culture was used for the extraction of the genome. According to the Bacteriological Analytical Manual (BAM), the cell number was calculated using plate count agar (Hopebio, Qingdao, China).

The total DNA was extracted using a DNA extraction kit (Bioteke Corporation, Beijing, China) in accordance with the manufacturer’s instructions. The concentration and quality of the DNA were measured using a spectrophotometer (DU730, Beckman Coulter, Burea, CA, USA). The genomic DNA was stored at −20 °C until use.

### 2.2. Primer Design

Primers and probes were designed on the basis of the *hlyA* gene (Genebank accession: HM58959) of *Listeria monocytogenes*, *toxR* gene (Genebank accession: GQ228073.1) of *Vibrio parahaemolyticus*, and *rfbE* gene (Genebank accession: AE005429) of *Escherichia coli O157:H7*. In addition, primers were designed according to RPA guidelines from TwistDx. The primers (Table 2) with target-specific labels were tagged with cyanine 5 (Cy5) and digoxin (primers for the *hlyA* gene), biotin and digoxin (primers for the *toxR* gene), or carboxy fluorescein (FAM) and digoxin (primers for the *rfbE* gene). The primers of *Vibrio parahaemolyticus* were obtained from our previous work [25].

### 2.3. Multiplex Reaction Protocols for RPA

RPA was performed in a 50 µL volume with a TwistAmp Basic Kit (TwistDX, Cambridge, UK). Briefly, each reaction mixture contained 25 µL of 2× reaction buffer, 15 µL of nuclease-free water, 2 µL each of specific primers (10 µM) for *Listeria monocytogenes*, *Vibrio parahaemolyticus*, and *Escherichia coli O157:H7*, 0.5 µL of each template and a dried enzyme pellet, and 2.5 µL of 14 mM magnesium acetate. Then, the reaction mixture was immediately incubated at 37 °C for 20 min.

### 2.4. Preparation of EuNP Probes

First, the conjugation process of EuNP-labeled anti-digoxin monoclonal antibody (EuNPs–anti-digoxin mAb) was performed [26]. Two milligrams of carboxylic EuNPs was dissolved in 800 μL of 2-(N-morpholino) ethanesulfonic acid (MES, 0.05 M, pH 8.2), before adding 30 μL of 1-(3-dimethylaminopropyl)-3-ethylcarbodiimide hydrochloride (EDC) to activate. After 30 min of incubation with slow shaking, the activated solution was centrifuged at 12,000 rpm for 25 min to separate the EDC. Then, 1 mL of 10 μg/mL anti-digoxin monoclonal antibody (anti-digoxin mAb) was added and stirred gently at 25 °C for 2 h. The conjugation was centrifuged for 2 min at 12,000 rpm to separate unreacted antibodies. Finally, the precipitates were resuspended in 1 mL of the preservation solution and stored at 4 °C until use.

### 2.5. Preparation of EuNP-Based LFIA and CG–LFIA

**Figure 1:**
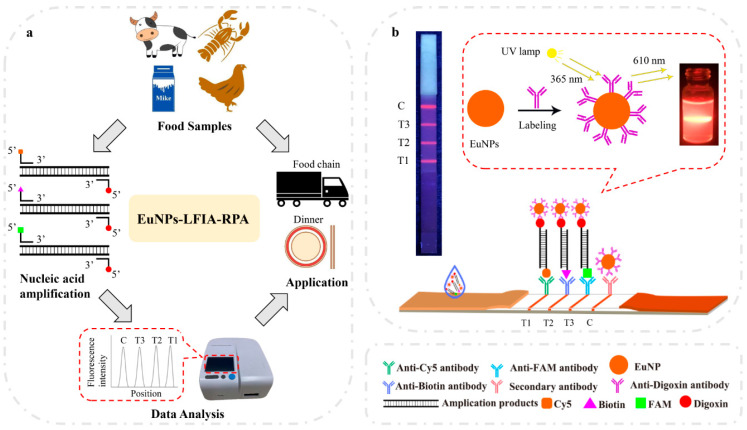
Schematic of EuNP-based LFIA–RPA. The application (**a**) and working principle (**b**) of the EuNP-based recombinase polymerase amplification combined with lateral flow immunoassay (EuNP-based LFIA–RPA).

A sample pad, a nitrocellulose filter (NC) membrane, an absorbent pad, and a backing card are the four elements of a lateral flow immunoassay (Figure 1). The EuNPs–anti-digoxin mAb conjugates were sprayed onto the conjugate pad. The anti-Cy5 antibody, anti-biotin antibody, and anti-FAM antibody were sprayed onto the three test lines to detect *Listeria monocytogenes*, *Vibrio parahaemolyticus*, and *Escherichia coli O157:H7.* The three test lines were located on the NC membrane. The immobilized goat anti-mouse polyclonal antibody (pAb) on the control line served as an assay control. The sample pads were immersed in phosphate-buffered saline (PBS, 0.01 M, pH7.4). Then, the pads were dried at 37 °C for 12 h and stored in a sealed bag at room temperature until use. For CG–LFIA, the conjugation of colloidal gold and anti-digoxin antibody was sprayed onto the conjugate pad, while the remaining conditions were the same as for the EuNP-based LFIA.

### 2.6. Multiplex EuNP-Based LFIA–RPA Assay

Firstly, multiplex RPA amplification was performed to produce labeled DNA amplification products. Then, a 50 μL sample solution was added dropwise onto the sample pad and diverted to the other end of the strip by capillary force. After a reaction time of 10 min, the result was observed under a portable 365 nm ultraviolet (UV) lamp. Simultaneously, the test strips were placed into a test strip reader, and the fluorescence intensities of the T1, T2, T3, and C lines were recorded. An FIC-S2011-B14 fluorescent strip reader (Suzhou Helmen Precise Instruments, Suzhou, Jiangsu, China) was used to scan the strip results. For CG–LFIA–RPA, the results were similarly obtained using a strip reader (TSR-200, Allsheng Instruments Co. Ltd., Hangzhou, China).

### 2.7. Optimization of the Multiplex EuNP-Based LFIA–RPA Assay

In order to optimize the multiplex EuNP-based LFIA–RPA assay, different primer concentrations were used. On the basis of preliminary experiments, it was found that the primer concentration for *Vibrio parahaemolyticus* of 150 nM had the highest amplification efficiency. To achieve a similar amplification efficiency, the reaction used the 150 nM primer concentration of *Vibrio parahaemolyticus* and a gradient of 150, 200, 250, 300, 350, and 400 nM primer concentrations for *Listeria monocytogenes* and *Escherichia coli O157:H7*. Then, seven distinct reaction times (2.5, 5, 10, 15, 20, 25, and 30 min) were compared for EuNP-based LFIA. The experiments were repeated with CG–LFIA. To realize the optimal conditions, the amounts of the EuNP conjugate and the concentrations of the three antibodies on the T lines were optimized using cross-reaction experiments. For *Listeria monocytogenes*, *Vibrio parahaemolyticus*, and *Escherichia coli O157:H7*, the concentrations of the antibodies ranged from 0.4 to 1.2 mg/mL, while the concentrations of the EuNP conjugate ranged from 2 to 8 ng/mL.

### 2.8. Sensitivity and Specificity of the Multiplex EuNP-Based LFIA–RPA Assay

Sensitivity and specificity experiments were performed for the optimized multiplex EuNP-based LFIA–RPA. In the sensitivity experiment, 10-fold serial dilutions of three food-borne strains were used to confirm the limits of detection (LODs). The dilutions of genomic DNA of *Listeria monocytogenes*, *Vibrio parahaemolyticus*, and *Escherichia coli O157:H7* ranged from 10^5^ to 10^0^ colony-forming units (CFU)/mL. The standard curves were constructed using a fluorescence reader. To determine the specificity of each primer set, the specificity experiment of the multiplex EuNP-based LFIA–RPA assay was assessed using DNA extracted from the 22 bacterial strains in Table 1. All tests were independently repeated thrice.

### 2.9. Stability of the Multiplex EuNP-Based LFIA–RPA Assay

For the stability study of the multiplex EuNP-based LFIA–RPA, fluorescent test strips were stored at room temperature in a dry and sealed manner. The test strips were tested at 1 week, 2 weeks, 1 month, 2 months, 3 months, and 6 months. *Listeria monocytogenes*, *Vibrio parahaemolyticus*, and *Escherichia coli O157:H7* contained 10^1^ CFU/mL for RPA amplification. After amplification, they were stored at –20 °C until use. Following reaction, the T/C value was recorded by the FIC-S2011-B14 fluorescent strip reader.

### 2.10. Analysis of Spiked Samples

The utility of the multiplex EuNP-based LFIA–RPA assay as a surveillance tool for detecting *Listeria monocytogenes*, *Vibrio parahaemolyticus*, and *Escherichia coli O157:H7* in food was evaluated. Food samples (beef, milk, chicken breast, and shrimp) were purchased from retail stores and stored at 4 °C. According to BAM, all samples were confirmed to be negative for *Listeria monocytogenes*, *Vibrio parahaemolyticus*, and *Escherichia coli O157:H7*. Then, 100 g of each meat was placed on a plate, covered with cling film, and incubated at 37 °C. After 24 h of incubation, 5 g of each meat sample was homogenized in 100 mL of 4 M NaCl for 30 s. On the other hand, 25 mL of raw milk was poured into an aseptic homogeneous cup with 225 mL of buffered peptone water (BPW) (10 g/L peptone, 5 g/L sodium chloride, 9 g/L disodium hydrogen phosphate dodecahydrate, 1.5 g/L potassium dihydrogen phosphate, pH 7.2) and thoroughly mixed by shaking. Each 1 mL sample of homogenate was contaminated with 10^3^, 10^2^, 10^1^, and 10^0^ CFU/mL. Then, 1 mL of each inoculated sample was collected for DNA extraction, by adding 10 mg/mL lysozyme (Bioteke Corporation, Beijing, China) at 37 °C for 5 min. Subsequently, 20 mg/mL proteinase K (Bioteke Corporation, China) was added to each sample at 60 °C for 15 min, to break the bacterial cell wall. According to the manufacturer’s protocol, the genomic DNA was extracted using a DNA extraction kit (Bioteke Corporation, China). Non-inoculated samples were used as the negative control.

### 2.11. Evaluation of Field Food Samples

Field food samples (beef, milk, chicken breast, and shrimp) were purchased from retail stores. Then, 25 g (or 25 mL) of each sample was added to 225 mL of BLEB, APW, and LST. The samples were mixed by swirling. Next, they were enriched at 37 °C by shaking at 200 rpm for 16 h. All samples were analyzed by EuNP-based LFIA–RPA, CG–LFIA–RPA, and BAM assays.

### 2.12. Data Analysis

The results of fluorescent EuNP-based LFIA and CG–LFIA were read using an FIC-S2011-B14 fluorescent strip reader and TSR-200 test strip reader, respectively. The standard curve of EuNP-based LFIA–RPA was plotted according to the concentration of genomic DNA and the T/C value. Data were analyzed using Microsoft Excel 2013 (Microsoft Inc., Washington, DC, USA).

## 3. Results

### 3.1. Establishment and Optimization of Multiplex EuNP-Based LFIA–RPA Assay

**Figure 2:**
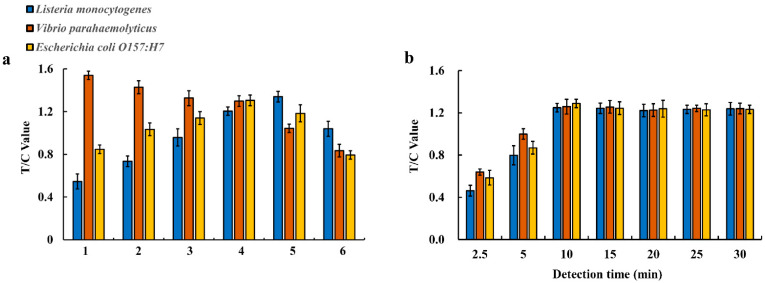
Optimization of the EuNP-based LFIA–RPA reaction. (**a**) Different primer concentrations of *Listeria monocytogenes*, *Vibrio parahaemolyticus*, and *Escherichia coli O157:H7*. 1: 150 nM (blue), 150 nM (orange), 150 nM (yellow); 2: 200 nM (blue), 150 nM (orange), 200 nM (yellow); 3: 250 nM (blue), 150 nM (orange), 250 nM (yellow); 4: 300 nM (blue), 150 nM (orange), 300 nM (yellow); 5: 350 nM (blue), 150 nM (orange), 350 nM (yellow); 6: 400 nM (blue), 150 nM (orange), 400 nM (yellow). Blue represents *Listeria monocytogenes*, orange represents *Vibrio parahaemolyticus*, and yellow represents *Escherichia coli O157:H7*. (**b**) Different detection times: 2.5 min, 5 min, 10 min, 15 min, 20 min, 25 min, and 30 min. The Y-axis represents the test line intensity (T/C value).

The principle of the multiplex EuNP-based LFIA–RPA assay is shown in Figure 1b. First, the labeled RPA amplification products were combined with the EuNPs–anti-digoxin mAb conjugates on the conjugate pad. Then, they were passed through the pad by capillary force. The three test lines on the NC membrane contained anti-Cy5 antibody, anti-biotin antibody, and anti-FAM antibody. The Cy5-, biotin-, and FAM-labeled duplex DNA was captured by the corresponding antibodies. Finally, the fluorescence intensity signal was detected by the FIC-S2011-B14 fluorescent strip reader. Several key factors, including the concentration of RPA primers, the detection time of LFIA, and the concentrations of the EuNP conjugate and the three antibodies on the test lines, were systematically optimized to obtain high sensitivity and fluorescence signals. The results show that the 150 nM primer concentration for *Vibrio parahaemolyticus* and the 300 nM primer concentration for *Listeria monocytogenes* and *Escherichia coli O157:H7* could obtain equivalent amplification efficiency (Figure 2a). After running the test strip, the T value and C value were recorded from 2.5 to 30 min. The T/C value displayed a continuous increasing trend (see Figure 2b) in the first 10 min of the reaction before reaching equilibrium. Thus, 10 min of detection time was considered necessary for all subsequent studies. The results of CG–LFIA–RPA are shown in Figure S1 (Supplementary Materials). According to the results, the optimal combinations of the concentrations of the EuNP conjugate and the antibodies on the T lines were as follows: 6 ng/mL of the EuNP conjugate and 0.8 mg/mL of anti-Cy5 antibody on the T1 line for *Listeria monocytogenes* detection; 6 ng/mL of the EuNP conjugate and 0.8 mg/mL of anti-biotin antibody on the T2 line for *Vibrio parahaemolyticus* detection; 6 ng/mL of the EuNP conjugate and 1.0 mg/mL of anti-FAM antibody on the T3 line for *Escherichia coli O157:H7* detection (Figure S2, Supplementary Materials).

### 3.2. Sensitivity of the Multiplex EuNP-Based LFIA–RPA

**Figure 3:**
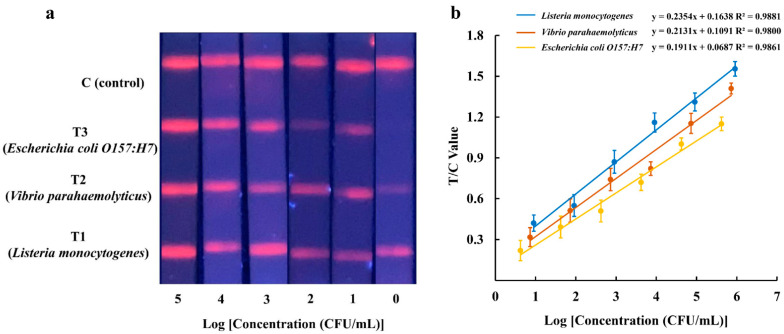
Reaction sensitivity of EuNP-based LFIA–RPA for *Listeria monocytogenes*, *Vibrio parahaemolyticus*, and *Escherichia coli O157:H7*. The amplified products could be observed using a portable 365 nm UV lamp (**a**). Standard curves for *Listeria monocytogenes*, *Vibrio parahaemolyticus*, and *Escherichia coli O157:H7* (**b**).

To evaluate the sensitivity of the multiplex EuNP-based LFIA–RPA assay, 10-fold serial dilutions of genomic DNA were prepared, and the purified bacterial genomic DNA was used as a template. The result was observed under a portable 365 nm UV lamp. As shown in Figure 3a, the fluorescence signals obtained from the EuNP-based LFIA–RPA assay were proportional to the dose of the three bacterial cultures in the range from 10^5^ to 10^0^ CFU/mL, indicating that this test produced a detectable amplicon with DNA concentrations as low as 10^0^ CFU/mL. Using the strip test reader to detect the intensity of the fluorescence signal on the test lines, it was possible to obtain quantitative results. The T/C value (fluorescence signal intensity) increased with a high concentration of the DNA template. The standard linear curves had the following correlation coefficients of determination: *Listeria monocytogenes*, R^2^ = 0.9881; *Vibrio parahaemolyticus*, R^2^ = 0.9800; *Escherichia coli O157:H7*, R^2^ = 0.9861 (Figure 3b). There was a significant correlation between the template concentration and the detection threshold. The results of CG–LFIA–RPA are shown in Figure S3 (Supplementary Materials). Compared with the CG–LFIA–RPA assay, the EuNP-based LFIA–RPA improved the LOD by up to 10 times.

### 3.3. Specificity of the Multiplex EuNP-Based LFIA–RPA

**Figure 4:**
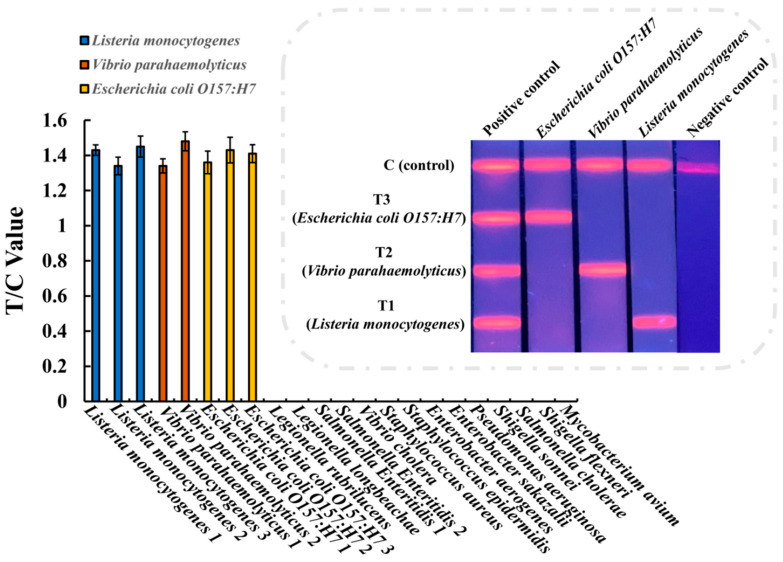
Specificity testing of EuNP-based LFIA–RPA. Specific evaluation results from 22 bacterial strains.

To determine the specificity of the assay, all 22 bacterial strains were investigated in the EuNP-based LFIA–RPA experiments, including 3 *Listeria monocytogenes*, 2 *Vibrio parahaemolyticus*, 3 *Escherichia coli O157:H7*, and 14 other food-borne strains. The genomic DNA of all 22 bacterial strains listed in Table 1 was extracted and added to the reaction to determine the specificity of the multiplex EuNP-based LFIA–RPA assay. The experimental results show that only the *Listeria monocytogenes*, *Vibrio parahaemolyticus*, and *Escherichia coli O157:H7* strains had positive results, while the other strains had negative results (Figure 4). There was no cross-reactivity among the *hlyA* gene for *Listeria monocytogenes*, *toxR* gene for *Vibrio parahaemolyticus*, and *rfbE* gene for *Escherichia coli O157:H7* during specificity testing.

### 3.4. Stability of the Multiplex EuNP-Based LFIA–RPA

The effect of the storage time of fluorescent test strips was examined by running 10^1^ CFU/mL amplification products of Listeria monocytogenes, Vibrio parahaemolyticus, and Escherichia coli O157:H7. The results are shown in Figure S4 (Supplementary Materials). In comparison with fresh fluorescent test strips, there were no obvious changes in the T/C value of Listeria monocytogenes and Vibrio parahaemolyticus, and Escherichia coli O157:H7 within 3 months. Additionally, there were no obvious changes in Listeria monocytogenes and Vibrio parahaemolyticus within 6 months. There was a significant decrease in the T/C value of Escherichia coli O157:H7 at 6 months. The results indicate that the fluorescent test strips were stable for at least 3 months at room temperature.

### 3.5. Application of the Multiplex EuNP-Based LFIA–RPA to Spiked Samples

To analyze how different samples affected the EuNP-based LFIA–RPA assay, the developed method was evaluated using beef, milk, chicken breast, and shrimp samples. The samples (beef, milk, chicken breast, and shrimp) were collected for *Listeria monocytogenes*, *Vibrio parahaemolyticus*, and *Escherichia coli O157:H7* detection to demonstrate the application of the EuNP-based LFIA–RPA assay. The samples were spiked with different concentrations of *Listeria monocytogenes*, *Vibrio parahaemolyticus*, and *Escherichia coli O157:H7*. The genomic DNA of each tested sample was extracted for EuNP-based LFIA–RPA and CG–LFIA–RPA detection. Table 3 shows that the quantitative detection results of spiked food for *Listeria monocytogenes*, *Vibrio parahaemolyticus*, and *Escherichia coli O157:H7* using the EuNP-based LFIA–RPA assay were similar to those obtained using CG–LFIA–RPA. The recoveries of *Listeria monocytogenes*, Vibrio parahaemolyticus, and *Escherichia coli O157:H7* in food samples were 90.9–114.2%, 92.4–113.8%, and 93.3–114.1%, respectively (Table 3).

### 3.6. Results of Field Food Samples

All 16 field food samples were tested using EuNP-based LFIA–RPA, CG–LFIA–RPA, and BAM assays (Table 4). The positive detection rates for *Listeria monocytogenes*, *Vibrio parahaemolyticus*, and *Escherichia coli O157:H7* were all 0%.

## 4. Discussion

Bacteria are tiny organisms which usually exist in the environment and food, including meat, fish, eggs, milk, and seafood. These bacteria contaminate food ingredients, making them harmful when eaten by causing gastrointestinal food-borne illnesses at any time during growth, harvesting, processing, storage, transportation, and preservation. *Listeria monocytogenes*, *Vibrio parahaemolyticus*, and *Escherichia coli O157:H7* are common food-borne pathogens that can cause various food-borne illnesses. Therefore, detection of these bacteria has important significance, especially in rapid testing. Several conventional techniques for the detection of pathogenic bacteria in food have been established. These methods include different biological detection techniques such as culture-dependent microbiological methods [27], polymerase chain reaction (PCR) [28,29], immunological detection techniques [30], and biosensor-based methods [31]. However, due to the need for specific equipment and the requirement of time-consuming procedures, these methods are not suitable for wide application in the field. Therefore, a number of isothermal amplification methods have been developed. Recently, loop-mediated isothermal amplification (LAMP), helicase-dependent amplification (HDA), and recombinase polymerase amplification (RPA) were developed as alternative methods to amplify nucleic acids [32,33]. Among these technologies, RPA has significant advantages, such as time efficiency, lower incubation temperature, thermal instrumentation independence, and strong tolerance of the sample matrix.

There are usually multiple pathogens in food; thus, traditional single-target nucleic acid detection can no longer meet the needs of multi-target detection. The ability to carry out multiple RPA amplifications in one reaction system has been successfully reported; for example, the RPA assay was used for the simultaneous detection of three food-borne pathogens in seafood and multiple bacteria in urine [25,34]. RPA combined with colloidal gold test strips has also been used, such as for the rapid detection of coliform bacteria using a lateral flow test strip assay [35], as well as in a dual fluorescein isothiocyanate (FITC) lateral flow immunoassay for the sensitive detection of *Escherichia coli O157:H7* in food samples [36]. However, there have been few reports on the fluorescent LFIA–RPA assay for the simultaneous detection of multiple food-borne pathogens. In this study, we described a quantitative and specific multiplex EuNP-based LFIA–RPA method for the accurate identification of three food-borne pathogens. This EuNP-based LFIA–RPA has potential practical applications for the detection of pathogens in food.

For the RPA reaction, the concentration of primers is an important factor for amplification efficiency. To achieve an equivalent amplification efficiency, we studied the influence of several sets of different concentration primers. The optimized results for primer concentrations were 300 nM for *Listeria monocytogenes*, 150 nM for *Vibrio parahaemolyticus*, and 300 nM for *Escherichia coli O157:H7*. Moreover, the optimal conditions for multiplex detection using the EuNP-based LFIA assay were determined to be 37 °C for 10 min. We found that the position of the T line of the multiple test strips could affect the detection efficiency. The T1 line is close to the sample pad and binds the EuNP–anti-digoxin mAb conjugate before the T2 and T3 lines, which is conducive to the reaction. The results of the T3 line for *Escherichia coli O157:H7* were weaker than those for *Listeria monocytogenes* and *Vibrio parahaemolyticus.* Therefore, these results can be considered for our optimization of the EuNP-based LFIA–RPA assay in the future.

Recently, many LFIA–RPA methods have been established, including recombinase polymerase amplification combined with a lateral flow strip for *Listeria monocytogenes* detection in food [5] and a dual FITC lateral flow immunoassay for sensitive detection of *Escherichia coli O157:H7* in food samples [36]. The EuNP-based LFIA–RPA assay can simultaneously detect as few as 10^0^ CFU/mL for *Listeria monocytogenes*, *Vibrio parahaemolyticus*, and *Escherichia coli O157:H7*, with the sensitivity showing a significant correlation among the colored signals. Compared with the colloidal gold detection method, the EuNP-based lateral flow immunoassay is a more sensitive method because EuNPs have unique photoluminescent properties, including a large Stokes shift, high emission fluorescence intensity, clear emission profile, and long emission fluorescence lifetime [24]. In this study, a fluorescent test strip reader was used to quantitatively analyze the results of the test strip. Furthermore, the delayed time-resolved mode of the strip reader eliminated the background autofluorescence noise and achieved a high signal-to-noise ratio. Accordingly, accurate intensity data of the fluorescent signal on the test lines can be read and obtained. This strip reader can, thus, reduce errors considered qualitative. When combined with a fluorescent test strip reader, the EuNP-based LFIA–RPA assay becomes a better method for detecting pathogenic bacteria. In addition, there was no cross-reaction with other bacteria. The results show that the method was highly specific. Additionally, the recovery rates of the spiked food samples were analyzed and compared with the colloidal gold method. The results show that the EuNP-based LFIA–RPA analysis had a higher recovery rate for the spiked food samples. In conclusion, the multiplex EuNP-based LFIA–RPA assay is a rapid and high-sensitivity detection method for food-borne pathogens.

## 5. Conclusions

In this study, a novel multiplex EuNP-based LFIA–RPA assay was established for the rapid detection of food-borne pathogens. The assay could simultaneously detect *Listeria monocytogenes*, *Vibrio parahaemolyticus*, and *Escherichia coli O157:H7*, and the strip reader allowed for a quantitative result. The multiplex EuNP-based LFIA–RPA assay is sensitive, specific, and time-efficient. In addition, this assay has potential application prospects in the field because the amplification could be completed in 20 min at 37 °C. In summary, the sensitive multiplex EuNP-based LFIA–RPA has practical significance for the rapid detection of food-borne pathogens in the field. In the future, the development of multiple quantitative test strip assays will become a powerful tool for monitoring multiple pathogens in food.

## Figures and Tables

**Table 1:** Information on bacterial strains used in this study.

Species	ID of Strains	Results of EuNP-Based LFIA–RPA
*Listeria monocytogenes*	ATCC19111	+
*Listeria monocytogenes*	ATCC19115	+
*Listeria monocytogenes*	ATCC7644	+
*Vibrio parahaemolyticus*	ATCC17802	+
*Vibrio parahaemolyticus*	ATCC33847	+
*Escherichia coli O157:H7*	GIMCC1.201	+
*Escherichia coli O157:H7*	ECO-071 *	+
*Escherichia coli O157:H7*	ATCC35150	+
*Legionella rubrilucens*	ATCC35304	−
*Legionella longbeachae*	ATCC33462	−
*Salmonella* Enteritidis	GIMCC1.345	−
*Salmonella* Enteritidis	ATCC13076	−
*Vibrio cholera*	GIMCC1.449	−
*Staphylococcus aureus*	ATCC25923	−
*Staphylococcus epidermidis*	SE-001 *	−
*Enterobacter aerogenes*	CICC10293	−
*Enterobacter sakazalii*	ATCC21550	−
*Pseudomonas aeruginosa*	GIMCC1.843	−
*Shigella sonnei*	GIMCC1.424	−
*Salmonella cholerae*	CICC21494	−
*Shigella flexneri*	CICC10865	−
*Mycobacterium avium*	CMCC93026	−

ID: identifier; EuNP: europium nanoparticle; LFIA: lateral flow immunoassay; RPA: recombinase polymerase amplification; GIMCC: Guangdong Microbiology Culture Center, Guangdong, China; ATCC: American Type Culture Collection, Virginia, United States of America (USA); CICC: China Center of Industrial Culture Collection, Shanghai, China; CMCC: National Center for Medical Culture Collections, Beijing, China. * Afforded by Zhejiang Academy of Science and Technology for Inspection and Quarantine; + positive result; − negative result.

**Table 2:** Sequences of RPA primers used for *Listeria monocytogenes*, *Vibrio parahaemolyticus*, and *Escherichia coli O157:H7*. CY5: cyanine 5; FAM: carboxy fluorescein; FP: forward primer; RP: reverse primer.

Target Name	Primer Name	Sequence (5′–3′)	Modifications	Amplification Size
*Listeria monocytogenes* (*hlyA*)	RPA-FP	CGATCACTCTGGAGGATACGTTGCTCAATT	5′-Cy5	154 bp
	RPA-RP	TTACCAGGCAAATAGATGGACGATGTGAAA	5′-Digoxin	
*Vibrio parahaemolyticus* (*toxR*)	RPA-FP	TTTGTTTGGCGTGAGCAAGGTTTTGAGGTG	5′-Biotin	230 bp
	RPA-RP	GCAGAGGCGTCATTGTTATCAGAAGCAGGT	5′-Digoxin	
*Escherichia coli O157:H7* (*rfbE*)	RPA-FP	TATCTGCAAGGTGATTCCTTGATGGTCTCA	5′-FAM	176 bp
	RPA-RP	AGGCCAGTTACCATCCTCAGCTATAGGGTG	5′- Digoxin	

**Table 3:** Determination results for *Listeria monocytogenes*, *Vibrio parahaemolyticus*, and *Escherichia coli O157:H7* in spiked samples. CFU: colony-forming unit.

Sample (*n* = 8 Each)	*Listeria monocytogenes*			*Vibrio parahaemolyticus*			*Escherichia coli O157:H7*		
	Spiked Concentration (CFU/mL)	Recovery of EuNP-Based LFIA (%)	Recovery of CG–LFIA (%)	Spiked Concentration (CFU/mL)	Recovery of EuNP-Based LFIA (%)	Recovery of CG–LFIA (%)	Spiked Concentration (CFU/mL)	Recovery of EuNP-Based LFIA (%)	Recovery of CG–LFIA (%)
Beef	9.0 × 10^5^	105.5	104.2	7.0 × 10^5^	110.3	105.7	4.0 × 10^5^	113.5	111.1
	9.0 × 10^4^	103.3	103.8	7.0 × 10^4^	110.0	103.2	4.0 × 10^4^	113.2	110.4
	9.0 × 10^3^	101.7	101.5	7.0 × 10^3^	108.5	101.4	4.0 × 10^3^	112.4	107.5
	9.0 × 10^2^	99.5	98.9	7.0 × 10^2^	103.7	100.6	4.0 × 10^2^	107.5	101.8
	9.0 × 10^1^	93.1	93.7	7.0 × 10^1^	99.2	94.1	4.0 × 10^1^	99.6	96.8
	9.0 × 10^0^	90.9	/	7.0 × 10^0^	92.4	/	4.0 × 10^0^	93.6	/
Milk	9.0 × 10^5^	114.2	110.4	7.0 × 10^5^	113.6	103.2	4.0 × 10^5^	110.5	106.2
	9.0 × 10^4^	114.0	110.1	7.0 × 10^4^	112.8	103.6	4.0 × 10^4^	109.4	106.0
	9.0 × 10^3^	112.9	108.3	7.0 × 10^3^	111.2	101.5	4.0 × 10^3^	108.8	104.4
	9.0 × 10^2^	110.4	104.3	7.0 × 10^2^	112.0	99.5	4.0 × 10^2^	109.1	102.7
	9.0 × 10^1^	97.9	96.1	7.0 × 10^1^	98.6	94.7	4.0 × 10^1^	94.5	94.6
	9.0 × 10^0^	91.3	/	7.0 × 10^0^	93.7	/	4.0 × 10^0^	93.3	/
Chicken breast	9.0 × 10^5^	112.1	106.4	7.0 × 10^5^	111.4	113.2	4.0 × 10^5^	114.1	112.8
	9.0 × 10^4^	111.4	104.8	7.0 × 10^4^	109.8	113.0	4.0 × 10^4^	113.8	110.4
	9.0 × 10^3^	110.5	105.1	7.0 × 10^3^	108.4	112.4	4.0 × 10^3^	113.5	111.1
	9.0 × 10^2^	108.1	103.2	7.0 × 10^2^	106.4	104.7	4.0 × 10^2^	106.5	106.4
	9.0 × 10^1^	93.8	100.3	7.0 × 10^1^	96.3	96.4	4.0 × 10^1^	96.8	99.9
	9.0 × 10^0^	92.4	/	7.0 × 10^0^	94.1	/	4.0 × 10^0^	93.9	/
Shrimp	9.0 × 10^5^	110.8	109.3	7.0 × 10^5^	113.8	106.3	4.0 × 10^5^	112.0	104.2
	9.0 × 10^4^	109.5	107.7	7.0 × 10^4^	113.2	104.9	4.0 × 10^4^	111.5	103.9
	9.0 × 10^3^	106.6	105.4	7.0 × 10^3^	112.6	103.3	4.0 × 10^3^	111.8	103.3
	9.0 × 10^2^	104.8	102.1	7.0 × 10^2^	109.0	101.1	4.0 × 10^2^	107.3	99.5
	9.0 × 10^1^	97.2	93.2	7.0 × 10^1^	98.1	93.1	4.0 × 10^1^	98.1	98.5
	9.0 × 10^0^	94.2	/	7.0 × 10^0^	93.4	/	4.0 × 10^0^	95.2	/

**Table 4:** Determination results for *Listeria monocytogenes*, *Vibrio parahaemolyticus*, and *Escherichia coli O157:H7* in field food samples.

Samples	*Listeria monocytogenes*			*Vibrio parahaemolyticus*			*Escherichia coli O157:H7*		
	EuNP-Based LFIA–RPA	CG–LFIA–RPA	BAM	EuNP-Based LFIA–RPA	CG–LFIA–RPA	BAM	EuNP-Based LFIA–RPA	CG–LFIA–RPA	BAM
BF-1	−	−	−	−	−	−	−	−	−
BF-2	−	−	−	−	−	−	−	−	−
BF-3	−	−	−	−	−	−	−	−	−
BF-4	−	−	−	−	−	−	−	−	−
MK-1	−	−	−	−	−	−	−	−	−
MK-2	−	−	−	−	−	−	−	−	−
MK-3	−	−	−	−	−	−	−	−	−
MK-4	−	−	−	−	−	−	−	−	−
CB-1	−	−	−	−	−	−	−	−	−
CB-2	−	−	−	−	−	−	−	−	−
CB-3	−	−	−	−	−	−	−	−	−
CB-4	−	−	−	−	−	−	−	−	−
SP-1	−	−	−	−	−	−	−	−	−
SP-2	−	−	−	−	−	−	−	−	−
SP-3	−	−	−	−	−	−	−	−	−
SP-4	−	−	−	−	−	−	−	−	−

− negative result.

## Supplementary Materials

The following are available online at https://www.mdpi.com/article/10.3390/ijerph18094574/s1. Figure S1. Optimization of the CG–LFIA–RPA reaction. (a) Different primer concentrations of *Listeria monocytogenes*, *Vibrio parahaemolyticus*, and *Escherichia coli O157:H7*. 1: 150 nM (blue), 150 nM (orange), 150 nM (yellow); 2: 200 nM (blue), 150 nM (orange), 200 nM (yellow); 3: 250 nM (blue), 150 nM (orange), 250 nM (yellow); 4: 300 nM (blue), 150 nM (orange), 300 nM (yellow); 5: 350 nM (blue), 150 nM (orange), 350 nM (yellow); 6: 400 nM (blue), 150 nM (orange), 400 nM (yellow). Blue represents *Listeria monocytogenes*, orange represents *Vibrio parahaemolyticus*, and yellow represents *Escherichia coli O157:H7*. (b) Different detection times: 2.5 min, 5 min, 10 min, 15 min, 20 min, 25 min, and 30 min. The Y-axis represents the test line intensity (T/C value). Figure S2. Optimization of the EuNP-based LFIA–RPA reaction. (a) Effect of different concentrations of anti-Cy5 antibody and EuNP conjugate on the T/C value. (b) Effect of different concentrations of anti-biotin antibody and EuNP conjugate on the T/C value. (c) Effect of different concentrations of anti-FAM antibody and EuNP conjugate on the T/C value. Figure S3. Reaction sensitivity of CG–LFIA–RPA for *Listeria monocytogenes*, *Vibrio parahaemolyticus*, and *Escherichia coli O157:H7*. (a) The amplified products could be observed by the naked eye. (b) Standard curves for *Listeria monocytogenes*, *Vibrio parahaemolyticus*, and *Escherichia coli O157:H7*. Figure S4. Stability study results for *Listeria monocytogenes*, *Vibrio parahaemolyticus*, and *Escherichia coli O157:H7.* Data for day 0 come from 10^1^ CFU/mL in Figure 3b. * Represents *p* < 0.05: difference is significant at the 0.05 level (ANOVA *t* test).
