# Supplementary material for: A Rapid and Sensitive Europium Nanoparticle-Based Lateral Flow Immunoassay Combined with Recombinase Polymerase Amplification for Simultaneous Detection of Three Food-Borne Pathogens
Source: Int J Environ Res Public Health. 2021 Apr 26;18(9):4574. doi: 10.3390/ijerph18094574 (PMC8123443; doi:10.3390/ijerph18094574)
Supplement: Supplementary file 1 [file ijerph-18-04574-s001.zip › ijerph-1165523-supplementary.pdf]

Supplementary materials

For

A Rapid and Sensitive Europium Nanoparticle-Based Lateral Flow Immunoassay Combined with Recombinase Polymerase Amplification for Simultaneous Detection of Three Food-Borne Pathogens

Kai Chen, Biao Ma, Jiali Li, Erjing Chen, Ying Xu, Xiaoping Yu, Chuanxin Sun, Mingzhou Zhang

Supplementary Figures

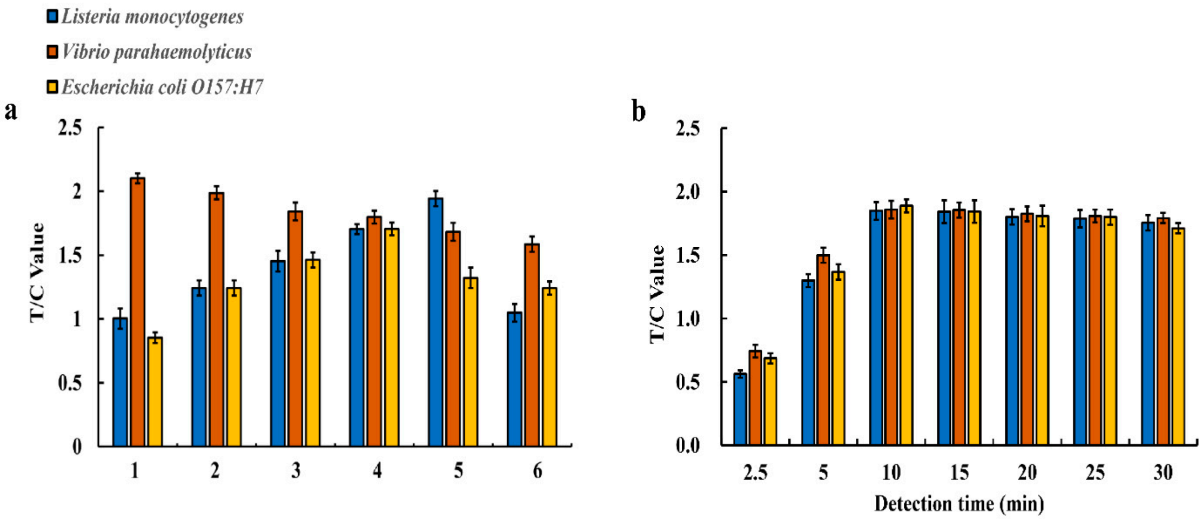

Figure S1. Optimization of the CG–LFIA–RPA reaction. **(a)** Different primer concentrations of *Listeria monocytogenes*, *Vibrio parahaemolyticus*, and *Escherichia coli O157:H7*, respectively. 1: 150 nM (blue), 150 nM (orange), 150 nM (yellow); 2: 200 nM (blue), 150 nM (orange), 200 nM (yellow); 3: 250 nM (blue), 150 nM (orange), 250 nM (yellow); 4: 300 nM (blue), 150 nM (orange), 300 nM (yellow); 5: 350 nM (blue), 150 nM (orange), 350 nM (yellow); 6: 400 nM (blue), 150 nM (orange), 400 nM (yellow). Blue represents *Listeria monocytogenes*, orange represents *Vibrio parahaemolyticus*, and yellow represents *Escherichia coli O157:H7*. **(b)** Different detection times: 2.5 min, 5 min, 10 min, 15 min, 20 min, 25 min, and 30 min. The Y-axis represents the test line intensity (T/C value).

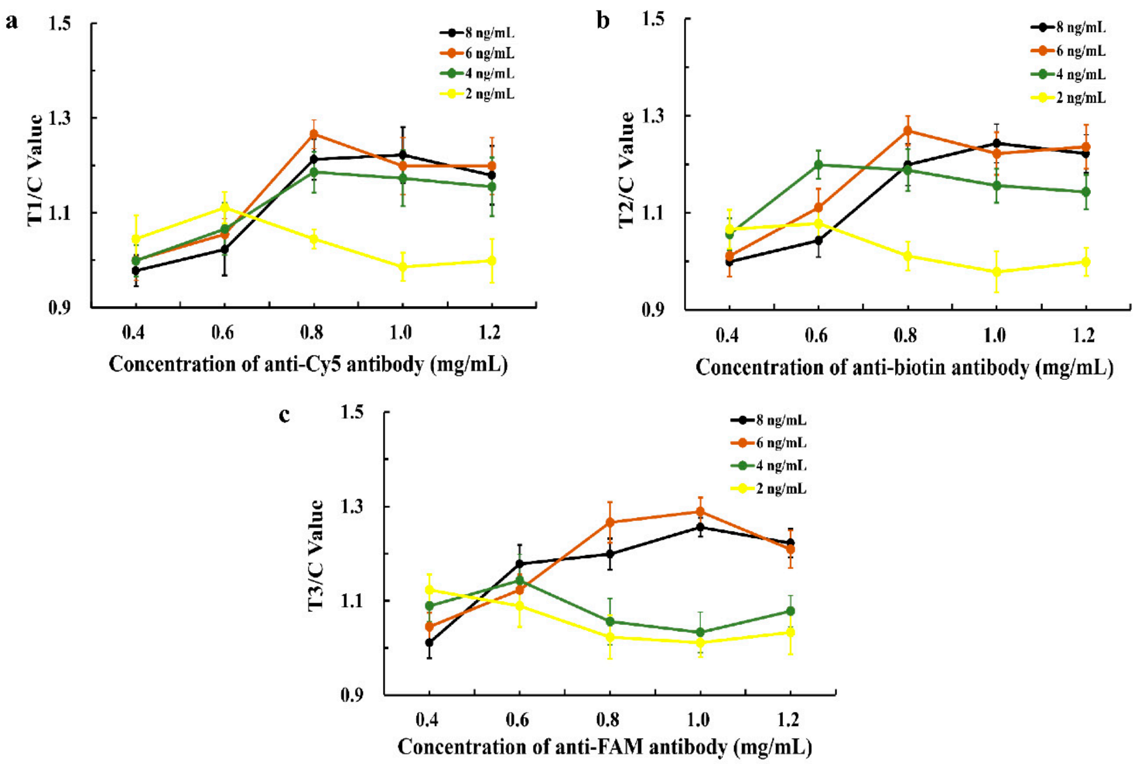

Figure S2. Optimization of the EuNP-based LFIA–RPA reaction. **(a)** Effect of different concentrations of anti-Cy5 antibody and EuNP conjugate on the T/C value. **(b)** Effect of different concentrations of anti-biotin antibody and EuNP conjugate on the T/C value. **(c)** Effect of different concentrations of anti-FAM antibody and EuNP conjugate on the T/C value.

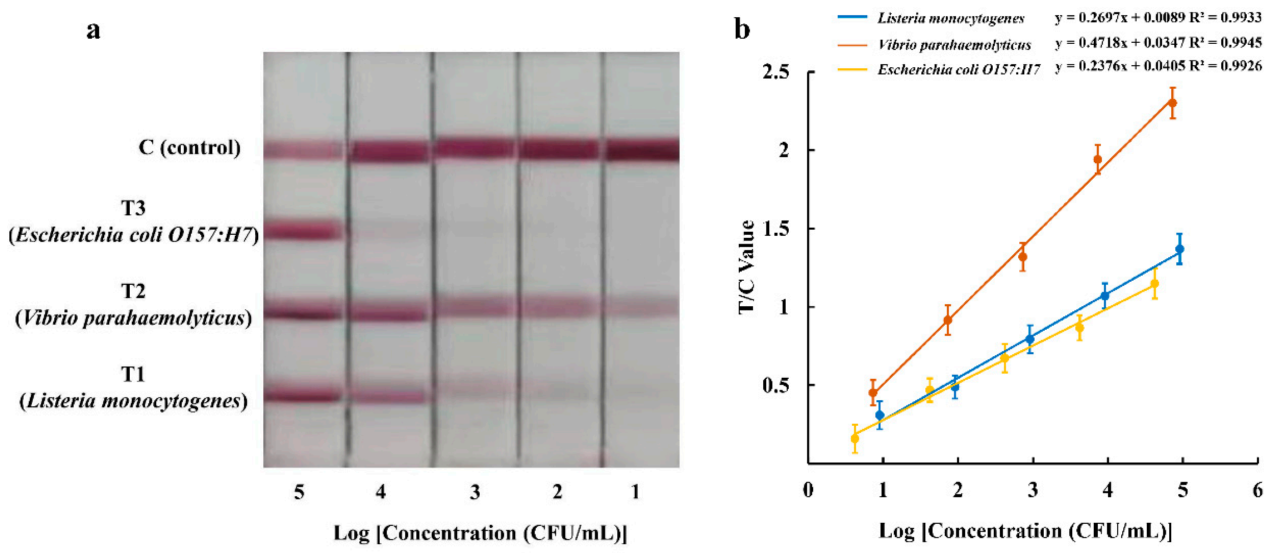

Figure S3. Reaction sensitivity of CG–LFIA–RPA for the *Listeria monocytogenes*, *Vibrio parahaemolyticus*, and *Escherichia coli O157:H7*. **(a)** The amplified products could be observed by the naked eye. **(b)** Standard curves for *Listeria monocytogenes*, *Vibrio parahaemolyticus*, and *Escherichia coli O157:H7*.

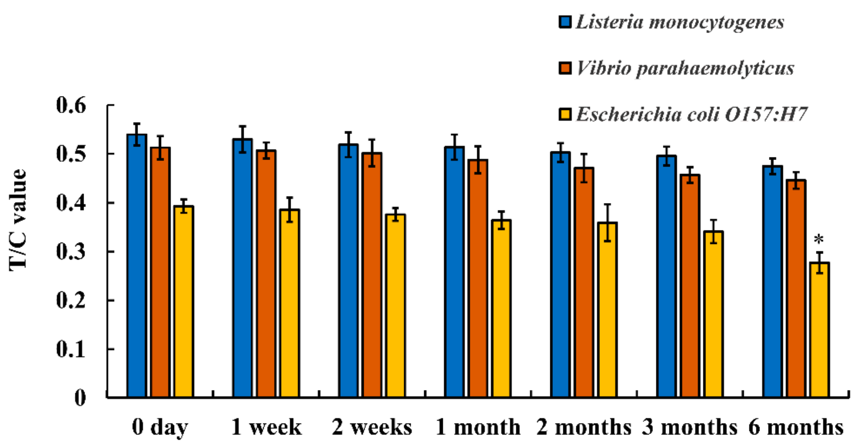

Figure S4. Stability study results for *Listeria monocytogenes*, *Vibrio parahaemolyticus*, and *Escherichia coli O157:H7*. Data for 0 day comes from 10^1 CFU/mL in Figure 3b. * Represent $p < 0.05$: difference is significant at the 0.05 level (ANOVA t test).
